# Evaluation of naturally acquired immune responses against novel pre-erythrocytic *Plasmodium vivax* proteins in a low endemic malaria population located in the Peruvian Amazon Basin

**DOI:** 10.1186/s12936-024-04978-z

**Published:** 2024-05-23

**Authors:** Julio A. Ventocilla, L. Lorena Tapia, Reynaldo Ponce, Adriano Franco, Mindy Leelawong, Joao C. Aguiar, G. Christian Baldeviano, Brandon K. Wilder

**Affiliations:** 1 Vysnova Partners Inc., Bethesda, USA; 2 U.S. Naval Medical Research Unit South, Lima-Peru (NAMRU SOUTH), Bellavista, Peru; 3 Instituto Nacional de Salud (INS), Lima, Peru; 4 Johns Hopkins University, Baltimore, USA; 5 CAMRIS International, LLC, Bethesda, USA; 6 Oregon Health & Science University, Portland, USA; 7 Universidad Peruana Cayetano Heredia, Lima, Peru; 8 Present Address: NYC Department of Health and Mental Hygiene, Long Island City, USA; 9 Present Address: Bluebird Bio, Inc, Somerville, USA

**Keywords:** *Plasmodium vivax*, Pre-erythrocytic stage, Natural acquired immunity, Peruvian amazon Basin

## Abstract

**Background:**

*Plasmodium vivax* represents the most geographically widespread human malaria parasite affecting civilian and military populations in endemic areas. Targeting the pre-erythrocytic (PE) stage of the parasite life cycle is especially appealing for developing *P. vivax* vaccines as it would prevent disease and transmission. Here, naturally acquired immunity to a panel of *P. vivax* PE antigens was explored, which may facilitate vaccine development and lead to a better understanding of naturally acquired PE immunity.

**Methods:**

Twelve *P. vivax* PE antigens orthologous to a panel of *P. falciparum* antigens previously identified as highly immunogenic in protected subjects after immunization with radiation attenuated sporozoites (RAS) were used for evaluation of humoral and cellular immunity by ELISA and IFN-γ ELISpot. Samples from *P. vivax* infected individuals (n = 76) from a low endemic malaria region in the Peruvian Amazon Basin were used.

**Results:**

In those clinical samples, all PE antigens evaluated showed positive IgG antibody reactivity with a variable prevalence of 58–99% in recently *P. vivax* diagnosed patients. The magnitude of the IgG antibody response against PE antigens was lower compared with blood stage antigens MSP1 and DBP-II, although antibody levels persisted better for PE antigens (average decrease of 6% for PE antigens and 43% for MSP1, p < 0.05). Higher IgG antibodies was associated with one or more previous malaria episodes only for blood stage antigens (p < 0.001). High IgG responders across PE and blood stage antigens showed significantly lower parasitaemia compared to low IgG responders (median 1,921 vs 4,663 par/µl, p < 0.05). In a subgroup of volunteers (n = 17),positive IFN-γ T cell response by ELISPOT was observed in 35% vs 9–35% against blood stage MSP1 and PE antigens, respectively, but no correlation with IgG responses*.*

**Conclusions:**

These results demonstrate clear humoral and T cell responses against *P. vivax* PE antigens in individuals naturally infected with *P. vivax.* These data identify novel attractive PE antigens suitable for use in the potential development and selection of new malaria vaccine candidates which can be used as a part of malaria prevention strategies in civilian and military populations living in *P. vivax* endemic areas.

## Background

*Plasmodium* spp. are the causative agent of malaria, one of the world’s deadliest infectious diseases. In 2022, about 249 million cases of malaria were reported by WHO with approximately 608,000 deaths, particularly in young children and pregnant women in sub-Saharan Africa [1]. While *Plasmodium falciparum* is the most prevalent malaria parasite on the African continent and responsible for most observed infections and deaths, *P. vivax* represents the most widely distributed human malaria parasite worldwide. Outside of Africa, *P. vivax* is the dominant cause of malaria with over 3 billion people living within its transmission limits [1]. Globally, several million clinical cases of *P. vivax* malaria are detected each year with a relevant portion in South East Asia and Latin America, where *P. vivax* is responsible for 50% and 70–85% of all malaria cases, respectively [1]. Traditionally, *P. vivax* has been considered to cause a “benign” form of malaria but is now recognized as a significant cause of morbidity and mortality due to increasing evidence of severe cases with a possible fatal outcome [2].

Malaria elimination efforts have resulted in a substantial decline in the global malaria burden in the past two decades. It is estimated that these efforts have resulted in a reduction of global malaria infections by 29% and mortality caused by malaria by 60% between 2000 and 2018 [1]. However, from 2014 to 2018 this downward trend has slowed considerably and even reversed in some regions (e.g., Venezuela) [1, 3]. *Plasmodium vivax* represents a special challenge to control efforts due to its unique biological features, including low-density blood-stage infection, asymptomatic infections and the formation of hypnozoites—the dormant forms residing in hepatocytes that can cause relapses months or years after the initial infection. Hypnozoites are believed to be responsible for up to 80% of infections and there is currently no diagnostic tool available for their detection [4]. Importantly, activation of hypnozoites can represents a big challenge for malaria elimination, especially in naïve civilian and military populations which travel to these *P. vivax* endemics zones. Previous studies showed relapse episodes in U.S. military population deployed to Afghanistan, where 38 Army Rangers reported activation of hypnozoites within an average of 233 days after return from *P. vivax* endemic areas, highlighting the risk to military personnel during deployments [5]. Further progress towards malaria elimination will require additional tools including the development of an effective vaccine or intervention, which can prevent or eliminate these dormant stages.

For many years, the efforts to develop a PE malaria vaccine have mainly been focused on *P. falciparum* while only two *P. vivax* vaccine candidates have reached clinical trials, targeting blood stage proteins DBPII and MSP-1 [5]. The most advanced and recently approved vaccine by WHO is the *P. falciparum* subunit vaccine RTS,S-AS01, which targets the Circumsporozoite Protein (CSP) via neutralizing antibodies and has shown consistent but short-lived efficacy of 25–36% in children in phase III and IV clinical trials [6, 7]. A novel R21 vaccine candidate with a similar target and vaccine platform as RTS,S/AS01 but with a significant increased ratio of CSP protein in vaccine formulation showed higher efficacy of 67–78% over 6–18 months children in clinical Phase I-III [8–10]. In addition to CSP, other proteins have been under investigation as vaccine targets such as the PE antigen thrombospondin-related adhesive protein (TRAP) and the highly promising blood stage protein Reticulocyte-binding Protein Homolog 5 (Rh5) [11]. Both candidates were chosen at least in part based on data showing that responses were associated with protection in naturally infected persons, currently Rh5 is progressing into phase I and II clinical trials after promising preclinical results in non-human primates [12–14].

Only a few such studies of naturally acquired immunity to *P. vivax* have been conducted, largely with a focus on antibodies to erythrocytic antigens. In these studies, reticulocyte-binding proteins critical for merozoite invasion have been associated with protective immunity (e.g. *Pv*DBP-II and *Pv*RBP2b) [15–18]. Large-scale screenings have identified antibodies to other blood-stage antigens associated with protection particularly when seen in combinations rather than with any single antigen alone [19, 20]. However, even fewer studies have explored the natural immunogenicity of *P. vivax* PE antigens [21] despite the fact that the PE stages of *Plasmodium* can be targeted with both antibodies and T cells [22, 23]. For *P. falciparum*, a partially protective PE intervention that only results in a reduction of liver stage parasites will have no perceptible benefit to the person as even a small number of breakthrough parasites can yield fulminant blood stage disease. However, for *P. vivax*, preventing a portion liver stages would still not yield sterilizing protection against primary infection but would have proportional effects on elimination since the number of hypnozoites would be reduced, resulting in the reduced number of relapses that are the main driver of disease [4, 24]

Taken together, this suggests that antibodies and T cells targeting multiple PE *P. vivax* proteins could be an integral part of developing the first effective *P. vivax* vaccine. The almost exclusive focus on the blood stage has resulted in a general lack of *P. vivax* PE candidate antigens and an even more opaque understanding of their role in natural immunity or even simply immunogenicity. This paucity of data around PE immunity in *P. vivax* limits the ability to take the first steps towards novel vaccine candidates. Here, naturally acquired immunity to a panel of *P. vivax* PE antigens was explored as a key step to facilitate vaccine development and to better understand naturally-acquired PE *P. vivax* immunity.

## Methods

### Human plasma and lymphocyte samples

Human samples were obtained between 2012 and 2017 from three passive surveillance protocols approved by the Institutional Review Board of the U.S. Naval Medical Research Unit SOUTH (NAMRU SOUTH) in compliance with all applicable federal regulations governing the protection of human subjects (NMRCD.2007.0004, NMRCD.2010.0002 and NAMRU6.2012.0006).

Protocols NMRCD.2007.0004 and NAMRU6.2012.0006 were conducted in Iquitos, the largest city in the department of Loreto located in Peruvian Amazon Basin. Samples were collected from the Hospital Regional de Loreto (HRL, n = 41) and Hospital de Apoyo de Iquitos (HAI, n = 35). HRL is a specialized health centre for complex diseases whereas HAI attends patients with less complex symptomatology, giving basic treatments and rapid diagnostics for transmissible and non-transmissible diseases. Subjects presenting malaria symptoms (fever, headache, chills) at both health centres were invited to participate in the study and blood samples were collected after providing their consent. All subjects enrolled (n = 76) were diagnosed by microscopy using thick blood smear (counting number of parasites vs number white blood cells to determine parasite per μL of blood) and confirmed by PCR [25] for *P. vivax* mono-infection. Loreto is described as hypoendemic malaria area with co-endemicity of *P. vivax* and *P. falciparum*. *Plasmodium vivax* represents 80—90% of total malaria cases with an annual average incidence of 42,164 ± 7,779 cases during 2012–2017. In addition, *P. vivax* transmission is sustained and heterogeneous along the year [26], resulting in underestimated malaria prevalence due to low- parasitaemia and/or asymptomatic cases.

**Fig. 1:**
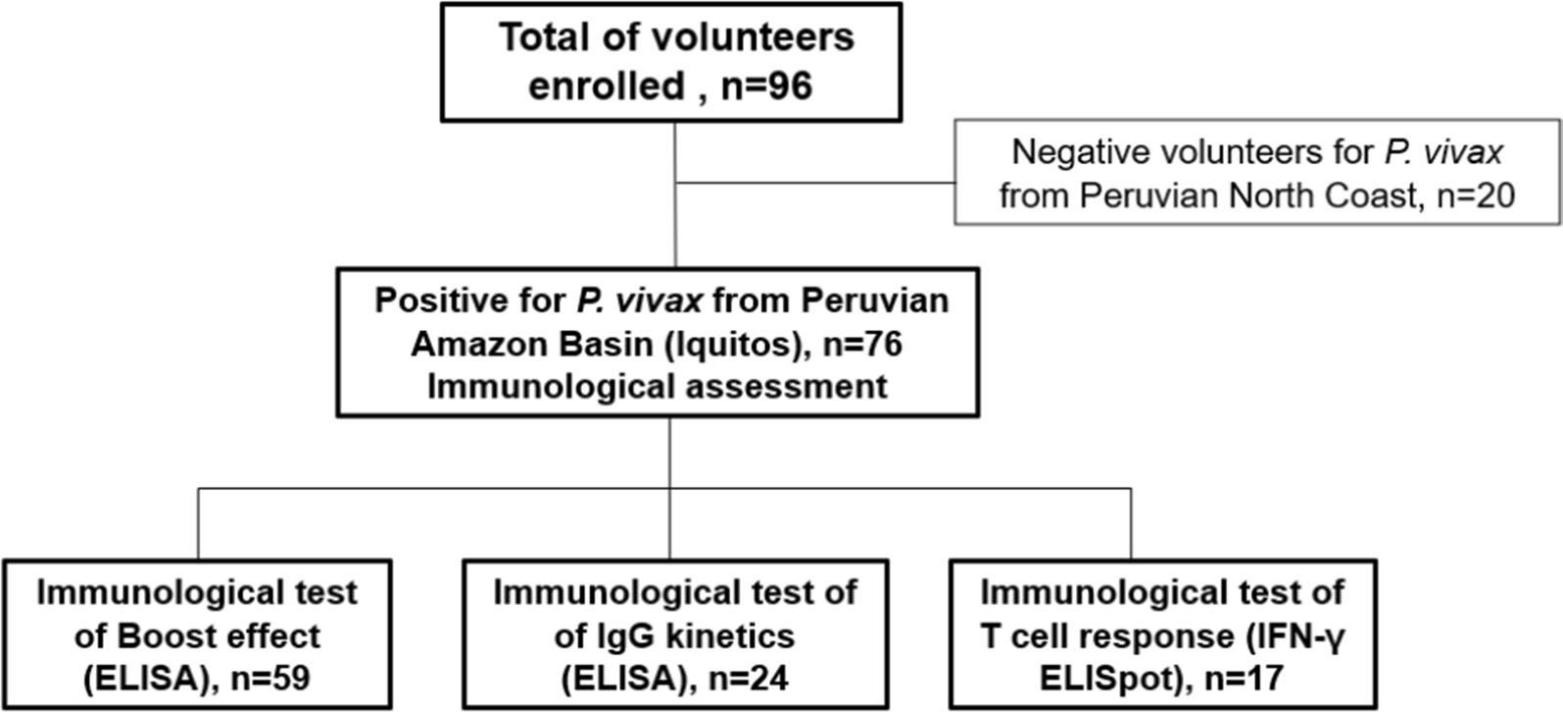
Enrolment Flow diagram. Description of volunteer enrolment and immunological procedures tested

In addition, a set of twenty human *P. vivax* negative control blood samples obtained from individuals living in the department of Piura was included, located in the North Coast of Peru (NMRCD.2010.0002), which is a very low incidence region for *P. vivax* (0.01 per 1000 inhabitants) during 2014 (Fig. 1).

### Selection of *P. vivax* PE antigens for immunological assessments

The 12 *P. vivax* PE antigens are orthologs to a panel of *P. falciparum* antigens previously identified as PE vaccine targets [27]. These *P. falciparum* antigens were selected from a larger set of 131 *P. falciparum* recombinant proteins based on their reactivity against sera samples from sterilely protected subjects who underwent immunization with radiation attenuated sporozoites (RAS) followed by challenge with the bites of viable *P. falciparum -*infected *Anopheles* mosquitoes [28, 29]. This approach has been partially validated for three *Plasmodium yoelii* orthologs from this list of candidates: a putative cysteine protease inhibitor (Falstatin), the gamete egress and sporozoite traversal (GEST) and the early transcribed membrane protein (ETRAMP) which induced up to 60% protective efficacy when used in combination using the DNA and vaccinia virus prime-boost immunization approach [30]. In addition, this subset of *P. vivax* proteins have been verified as expressed in the *P. vivax* proteome [31].

Proteins were expressed using the full open reading frame according to Plasmo DB using the *P. vivax* Sal-I reference genome, with predicted amino acid lengths between 90 and 500. Proteins were used if the predicted protein size between 12 and 65 kiloDaltons (kDa) was observed. These proteins were successfully expressed by the cell-free wheat germ expression system [32] using the bi-layer method (small-scale) with a yield between ~ 300-1200 μg of protein per reaction. In addition, well-known antigens MSP-1 (PVX_099980) and DBP-II (Sal-I) expressed at blood stage and CSP VK-210 (PVX_119355) expressed at PE stage were used to compare immunogenicity levels with novel PE orthologs.

### ELISA—human samples

Briefly, thirteen *P. vivax* PE antigens and two blood stage antigens were used to determine antibody prevalence and relative immunogenicity. ELISA plates were coated with recombinant proteins at concentration of 2–4 μg/ml to high-binding 96-well microplates (Nunc Maxisorp) overnight at room temperature and then washed with T-PBS1X (PBS1X/0.05%Tween20) five times. Microplates were blocked for 1 h with TBS1X-SM (TBS1X/Skim milk 5% buffer). After five washes with T-PBS1X, human plasma samples were added in duplicate at a 1:200 dilution in TBS1X-SM and incubated for 2 h at room temperature. After five washes with T-PBS1X, antibodies were detected using peroxidase-conjugated anti-human IgG monoclonal antibodies (Jackson Immunoresearch Cat#: 309–035-033, 1:6,000 dilution) and incubated for one hour at room temperature. After five washes with T-PBS1X, o-phenylenediamine (Sigma Aldrich Cat#: P3804) with hydrogen peroxide as a substrate was used and the reaction was stopped after 1 h of development with 50 μl of 3N HCl. Plates were read at 492 nm to determine optical density OD.

In order to assess variability between plates, a control on each plate a two-fold plasma serial dilution of a positive pool of 30 individuals with more than 10 *P. vivax* confirmed events was used. In addition, 20 plasma samples from individuals from malaria area with low incidence were used to calculate a positivity cut-off value for each protein using the average OD value plus 3 standard deviations.

### ELISpot

A library of overlapping 15-mer synthetic peptides was obtained from Mimotopes Pty Ltd. The peptides overlapped by 9 aminoacids spanning the entire blood and PE proteins. *Plasmodium vivax* MSP-1 was used as a positive control of blood stage antigens, and seven pre-erythrocytic antigens were tested (CSP, CelTOS, Falstatin, ETRAMP, PVX_119755, GEST and HSP PVX_089585). These were resuspended according to the manufacturer’s instructions and each peptide pool used at 10 μg/ml. These peptides were used to determine the frequency of T-cell response producing IFN-γ to *P. vivax* PE proteins using Human IFN-γ ELISpot PRO (Cat.# 3420-2APW-10. Mabtech AB, Sweden) according to the manufacturer’s instructions.

Peripheral blood mononuclear cells (PBMCs) from *P. vivax* patients, who accepted large blood collection for immunological assays, were isolated from whole blood by density centrifugation using a Percoll gradient, counted and cryopreserved for storage in liquid nitrogen. Briefly, PBMCs were thawed in RPMI media with 10% FBS (SIGMA, Cat #: F4135), incubated overnight at 37 °C and then plated at 0.2 × 10^6 cells/well in duplicates. Peptides were added at 10 μg/ml to stimulate T cell response for 18 h, in parallel phorbol 12-myristate 13-acetate/ionomycin (PMA/ION) without peptides was used as a positive stimulation control. Spots were counted using the CTL IMUNOSPOT Analyzer and values as number of spots over media-only controls. Subjects were defined as positive IFN- γ response when the number of spots was higher than the negative control (volunteers without previous malaria infection) over 20%.

### Statistical analysis

Variation of sample size per each analysis depended on the availability of samples, proteins quantity and data of epidemiological variables. Analysis was performed using STATA v16.0 statistical software (Stata Corp., College Station, TX, USA) and GraphPad Prism v9 (GraphPad Software, LLC). Differences among the frequency of epidemiological and immunological variables were analysed using Chi-square test for categorical variables and Mann–Whitney or Kruskal–Wallis test for numerical continuous variables to compare the median values and post-hoc Dunn’s test. Spearman rank correlation coefficient test was used to evaluate the correlation between epidemiological and immunological variables. To measure differences of antibodies level at different time points between groups (related samples) per each antigen, a paired Wilcoxon signed-rank test was used, the significance level for all statistical analysis performed was set at *p < *0.05* or **p < *0.001.*

## Results

### Epidemiological variables

Epidemiological variables by site of enrolment showed significant differences only for weight and number of previous *P. vivax* episodes (Table 1). Both variables were higher for patients enroled at Hospital Regional de Loreto (HRL). Therefore, samples from each location were analysed as a single group.

**Table 1:** Demographic information of *P. vivax* infected patients by site of sample collection

Characteristics	Hospital Apoyo Iquitos (HAI) n=35	Hospital regional Loreto (HRL) n=41	*p-value*
Age (Median, IQR)°	27	35	0.391
	(9–68)	(8–88)	
Male sex (%)"	54	60	0.309
Asexual par/ul (Median, IQR)°	2830	3045	0.692
	(36–14,130)	(24–24,180)	
Temperature C° (Median, IQR)°	37.8	37.0	0.198
	(36.0–41.5)	(35.9–39.6)	
Weight Kg (Median, IQR)°	56.0	65.0	0.037*
	(16–98)	(24–90)	
Number of previous	0	1	0.028*
*P. vivax* episodes (Median, IQR)°	(0–6)	(0–15)	

Bold values highlight the significance values for easy reading and interpretation of the readers.

Differences of epidemiological variables between groups of sample collection were assessed using Mann-Whitney U test (°) and Chi-square test (") with significant values for. *p < 0.05, and **p < 0.001. Hospital regional Loreto (HRL), Hospital Apoyo Iquitos (HAI)

### Human plasma samples from *P. vivax* infected population and humoral response

In order to determine if antibodies recognizing *P. vivax* PE antigens are induced during naturally occurring *P. vivax* infections, plasma samples from 76 *P. vivax* positive subjects enroled at health centres of HRL and HAI were tested during 2012–2017 by ELISA.

**Fig. 2:**
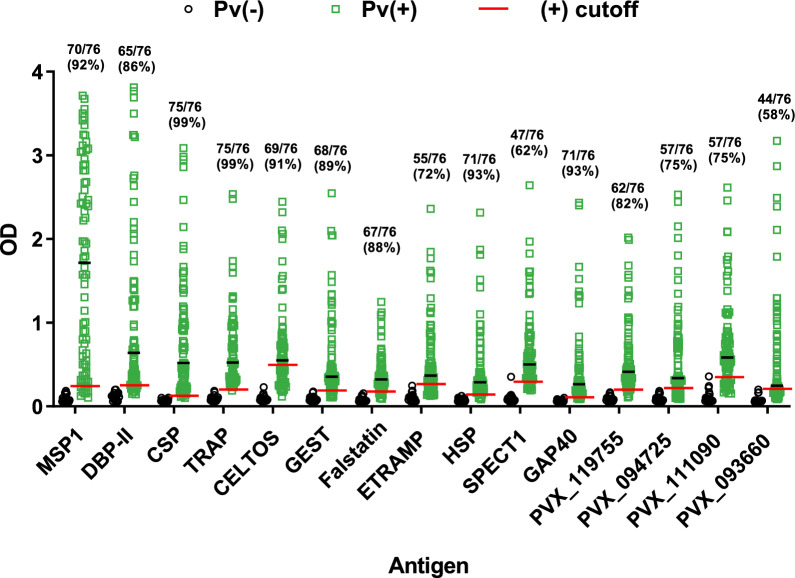
Prevalence against *P. vivax* PE and blood stage antigens in *P. vivax* infected patients. Plasma samples from healthy controls (black circles, n = 20) and *P. vivax* patients (green squares, n = 76) were tested against 15 *P. vivax* antigens to determine seroprevalence by ELISA. Data shows individual values of IgG antibodies against each antigen measured by OD values. OD positivity cut-off (red line) value was defined as the average of low endemic control samples plus three standard deviations per each antigen. Black line represents the average value of OD values per each antigen

This group of 76 *P. vivax* subjects showed positive antibody reactivity with high prevalence for blood stage antigens MSP1 (92%), DBP-II (86%) and the PE stage antigen CSP (99%) (Fig. 2). Twelve other *P. vivax* PE antigens showed positive antibody reactivity with variable prevalence in the range of 58–99%. Prevalence for each antigen was: TRAP PVX_082735 (99%), HSP PVX_089585 (93%), GAP40 PVX_080460 (93%), CELTOS PVX_123510 (91%), GEST PVX_118040 (89%), FALSTATIN PVX_099035 (88%), Hypothetical protein PVX_119755 (82%), Hypothetical protein PVX_094725 (75%), Hypothetical protein PVX_111090 (75%), ETRAMP (UIS3 ortholog) PVX_121950 (72%), SPECT1 PVX_083025 (62%), and Hypothetical protein PVX_093660 (58%) (Fig. 2).

The magnitude of the antibody response as measured by OD values was higher for the blood stage MSP1 antigen with an OD average of 1.8 compared to the canonical PE antigen CSP with OD average of 0.8. The other 12 *P. vivax* PE antigens showed variable intensity with a mean OD range of 0.3–0.7 (Fig. 2). Overall, the antibody magnitude did not correlate with parasitaemia levels at the time of sampling except for a modest negative correlation in four PE antigens (Spearman RHO: -0.23 to -0.28, p < 0.05) (Table 2). This indicates that variations in OD intensity for each antigen are likely a result of the intrinsic immunogenicity of each antigen and inter-individual differences in responses rather than differences due to the parasitaemia at time of enrolment.

**Table 2:** Correlation of parasitaemia levels with antibody response against blood and pre-erythrocytic *P. vivax* antigens in *P. vivax* infected patients

PE protein ID	PVX Code	Malaria stage	Asexual parasitemia par/ul vs antibodies OD	
			Rho Spearman	*p-value*
MSP1	PVX_099980	Blood	− 0.182	0.122
DBP-II/Sal-I		Blood	− 0.151	0.202
ETRAMP	PVX_121950	Pre-erythrocytic	− 0.289	0.012*
CSP	PVX_119355	Pre-erythrocytic	− 0.267	0.022*
SPECT1	PVX_083025	Pre-erythrocytic	− 0.255	0.029*
FALSTATIN	PVX_099035	Pre-erythrocytic	− 0.231	0.049*
CELTOS	PVX_123510	Pre-erythrocytic	− 0.204	0.082
Hypothetical	PVX_119755	Pre-erythrocytic	− 0.205	0.081
GEST	PVX_118040	Pre-erythrocytic	− 0.191	0.105
Hypothetical	PVX_111090	Pre-erythrocytic	− 0.176	0.135
HSP	PVX_089585	Pre-erythrocytic	− 0.168	0.154
GAP40	PVX_080460	Pre-erythrocytic	− 0.167	0.156
Hypothetical	PVX_094725	Pre-erythrocytic	− 0.075	0.524
TRAP	PVX_082735	Pre-erythrocytic	− 0.070	0.555
Hypothetical	PVX_093660	Pre-erythrocytic	− 0.008	0.944

Bold values highlight the significance values for easy reading and interpretation of the readers.

Plasma samples from *P. vivax* patients (n = 76) were tested against 15 *P. vivax* antigens to determine correlation between antibodies levels vs parasitaemia. Table shows Spearman Rho coefficient and *p-values* per each antigen.Significant values for *p< 0.05 and **p<0.001

**Fig. 3:**
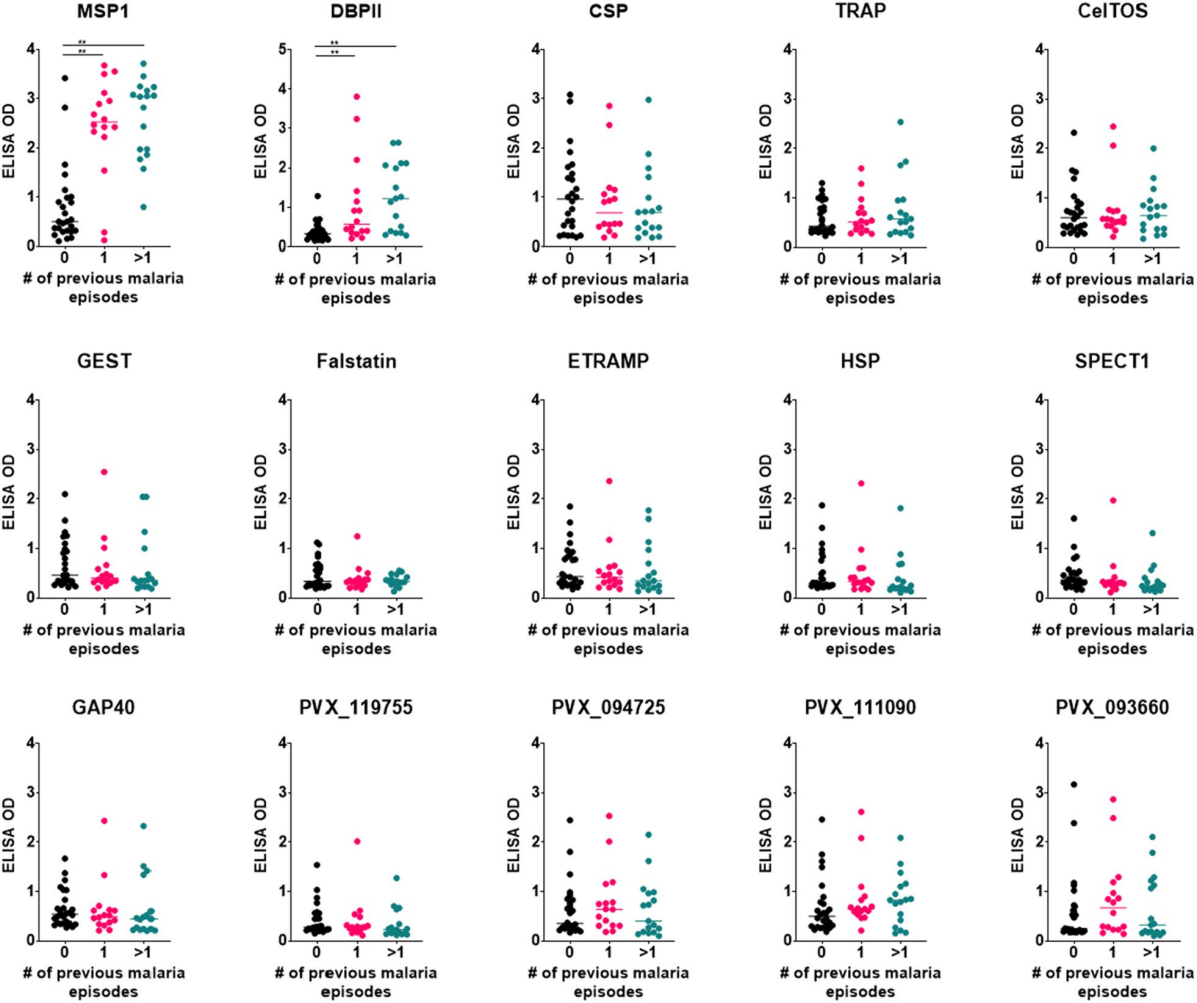
Boosting effect of blood and pre-erythrocytic *P. vivax* antigens in *P. vivax* infected patients. Plasma samples from zero previous (n = 26), one previous (n = 16) and two or more previous (n = 17) *P. vivax* episodes were used to determine boosting effect against each *P. vivax* antigen. Dot plots represent *P. vivax* episode groups with OD values of IgG antibodies against each antigen. Differences between groups per each antigen were assessed using the Kruskal–Wallis test with Dunn’s Post-hoc test. *p < 0.05, and **p < 0.001. Black, pink and green lines represent the average value of OD per each group of malaria episodes

To determine if antibody magnitude for each individual antigen correlated with previous malaria exposure, a subgroup of 59 patients from the total of 76 was used for which data of self-reported previous malaria episodes was available. Individuals were stratified by no previous (n = 26), one previous (n = 16) and two or more previous (n = 17) *P. vivax* episodes. There was a significant higher IgG antibodies against only the blood stage antigens MSP1 and DBP in groups with one or more previous episodes as compared to the group with no previous *P. vivax* infection (p < 0.05) (Fig. 3). IgG antibodies against all *P. vivax* PE antigens showed similar IgG levels independent of the number of self-reported previous malaria episodes (Fig. 3). Together, these data reveal a broad and variable seropositivity to multiple *P. vivax* PE antigens during acute *P. vivax* infection that, unlike blood stage antigens, appear not to be boosted by multiple blood stage infections.

**Fig. 4:**
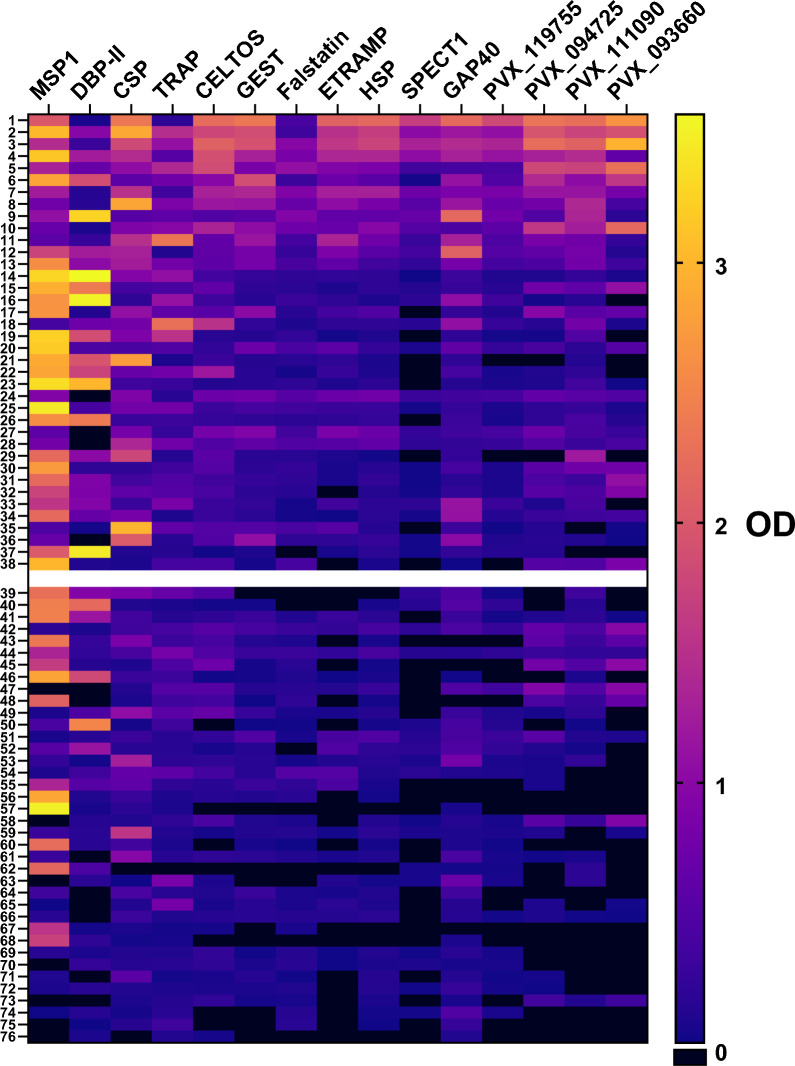
Reactivity of antibodies against blood stage and pre-erythrocytic *P. vivax* antigens in *P. vivax* infected patients. Individual reactivity against each *P. vivax* antigen is shown by OD. Negative reactivity of antibodies is shown as 0 (box dark blue colours) and positive reactivity of antibodies with OD values between 0.1 and 3.5 (boxes blue to yellow colours)

**Fig. 5:**
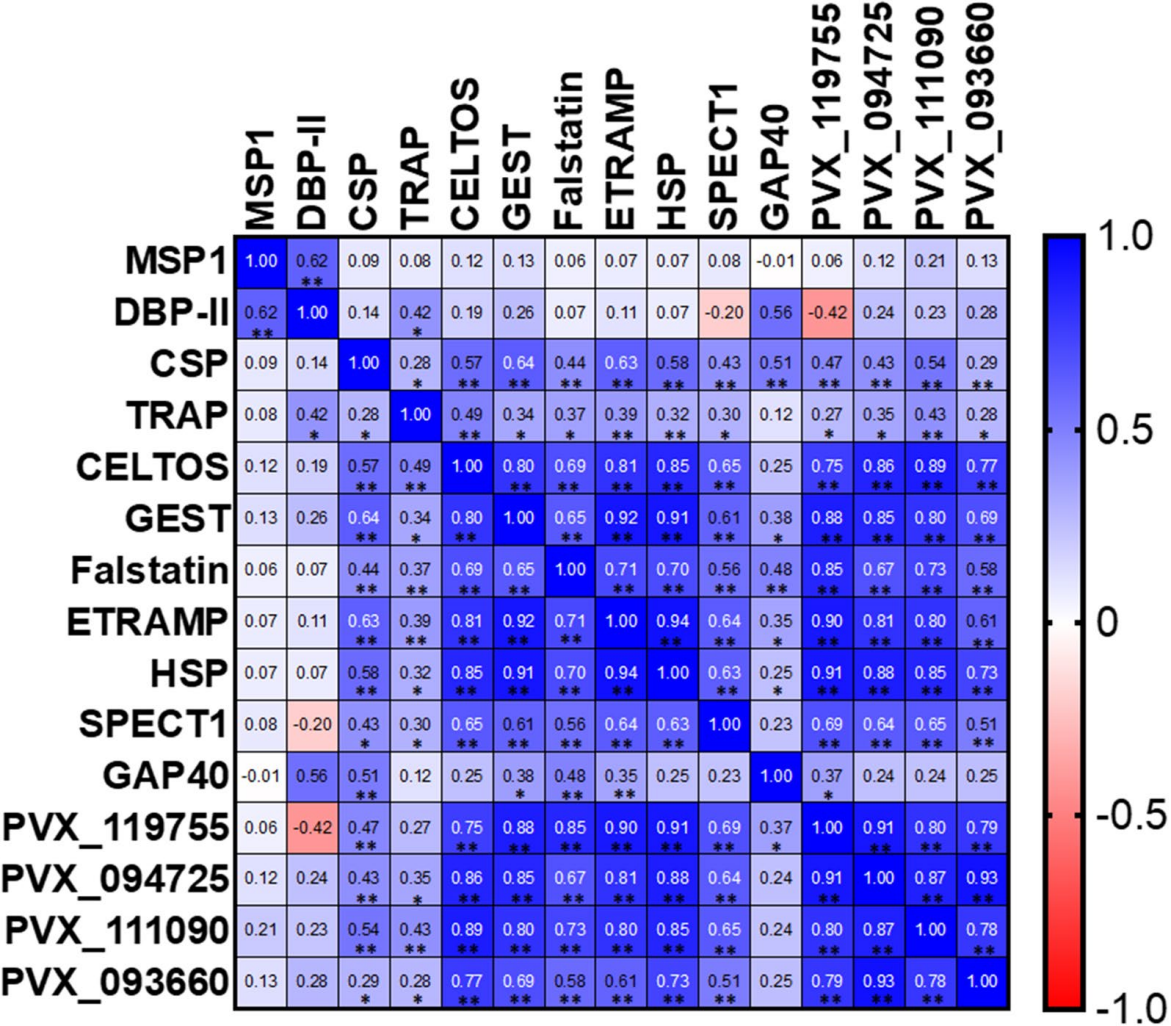
Correlation matrix of antibodies against blood and pre-erythrocytic antigens in *P. vivax* infected patients. Plasma samples from *P. vivax* infected volunteers were used to represent the correlation of antibody response between *P. vivax* antigens. Pearson’s correlation coefficients are shown with values of -1.0 to 1.0 with red indicating negative correlation and blue a positive correlation. Significant correlations were represented by *p < 0.05 and **p < 0.001

**Fig. 6:**
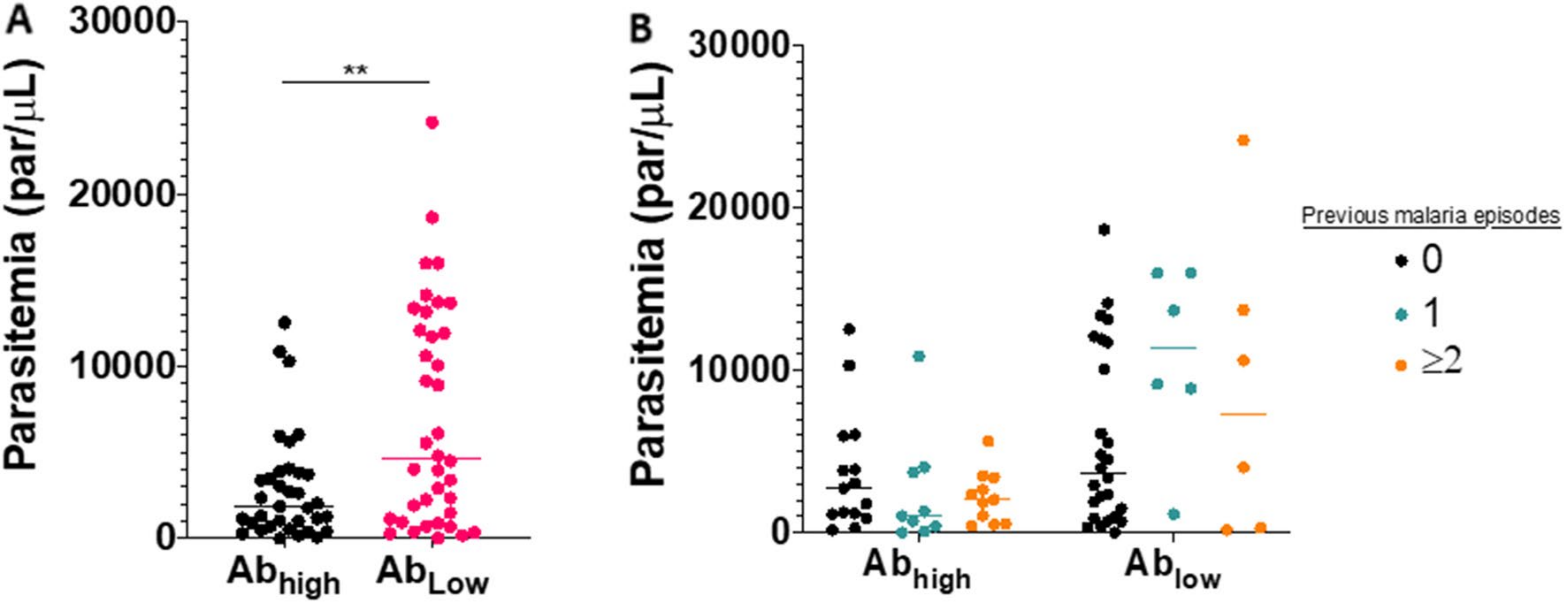
Relationship between parasitaemia and number of previous malaria episodes amongst high and low antibody responders in *P. vivax* infected patients. Plasma samples from *P. vivax* infected patients with high (n = 38) and low (n = 38) antibody response against blood and pre-erythrocytic antigens were used to evaluate association with parasitaemia levels. (A) Dot plots represent parasitaemia levels by high (black circles) and low (pink circles) antibody responders. (B) Dot plots represent parasitaemia levels in high and low antibody responder groups stratified by 0 (black circles), 1 (green circles) and ≥ 2 (orange circles) previous *P. vivax* episodes. Black, pink, green and orange lines represent average values of parasitaemia per groups. Significant differences are shown by *p < 0.05 and **p < 0.01

Antibody breadth (number of antigens for which a volunteer was positive) also showed substantial variability between volunteers, especially amongst pre-erythrocytic antibodies (Fig. 4). Some volunteers responded broadly to nearly every antigen while others appeared to have weak antibody responses in terms of both breadth and individual magnitude. Indeed, a common predictor of antibody response to one PE antigen was a response to another (Fig. 5) suggesting that some volunteers naturally respond to infection with a greater antibody response. Those “high responders”, defined simply as those in the top 50% of total IgG magnitude across all antigens were evaluated against epidemiological variables. Significant differences between high and low responders were not observed by place of sample collection, sex, age, weight, temperature, and number of previous *Pv* episodes. However, high responders showed a significantly lower parasitaemia compared to low responders (median par/µl 1921 vs 4663, p < 0.05) at time of enrolment (Table 3). Interestingly, high and low antibody responders did not differ in parasitaemia levels after stratification by number of previous episodes (Fig. 6A, B). Volunteers are arranged in order of descending total antibody response across all antigens with the gap showing the division between “high responders” (1–38) and “low responders” (39–76).

**Table 3:** Demographic information of *P. vivax* infected patients by low and high antibody responders

Characteristics	low Ab responders n=38	high Ab responders n=38	*p-value*	
Place-HRL (%)	44	56	0.250	
Place-HAI (%)	57	43	0.250	
Male sex (%)	49	51	0.817	
Age (Median, IQR)	30	30	0.790	
	(19–53)	(23–51)		
Asexual par/ul (Median, IQR)	4,663	1,921	0.014*	
	(36–24,180)	(24–12,572)		
Temperature C° (Median, IQR)	37.0	37.0	0.468	
	(36.0- 41.5)	(35.9–40.0)		
Weight Kg (Median, IQR)	60.0	66.5	0.433	
	(16-98)	(29–90)		
Number of previous	0	1	0.172	
*P. vivax* episodes (Median, IQR)	(0–8)	(0–15)		

Bold values highlight the significance values for easy reading and interpretation of the readers.

Epidemiological variables were compared between low and high responder group. Categorical variables were tested by Chi2 and numerical variables by Mann-Whitney U test. Significance was reported by *p < 0.05, and **p < 0.001. Hospital regional Loreto (HRL), Hospital Apoyo Iquitos (HAI)

**Fig. 7:**
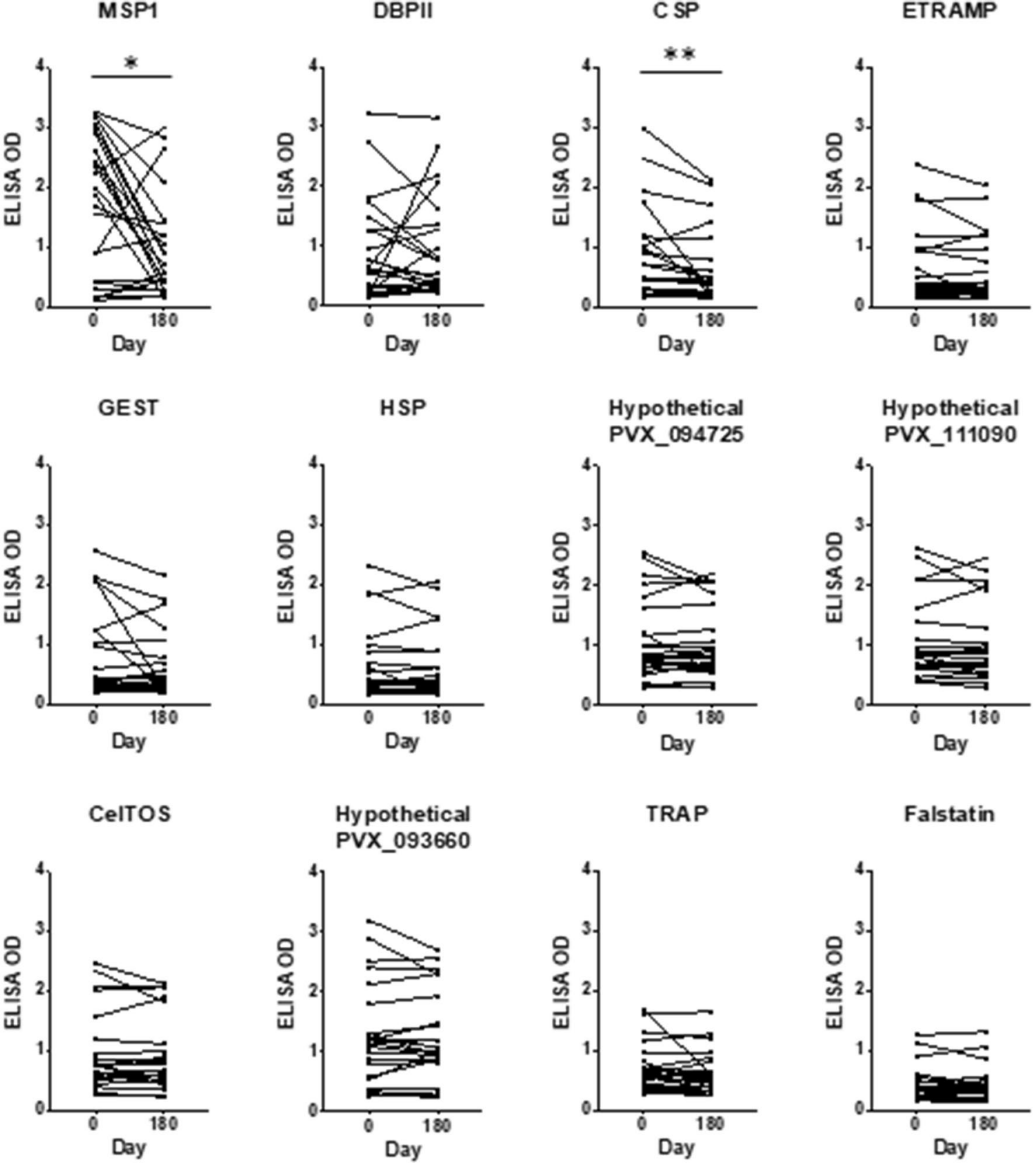
Long-term antibody analysis of blood and PE *P. vivax* antigens in *P. vivax* infected patients. Plasma samples from *P. vivax* infected patients (n = 24) were used to represent individual antibody variation measured by OD values after 6 months of *P. vivax* infection against each *P. vivax* antigen. Lines connect individual antibody variation between *P. vivax* infection Day 0 vs Day 180. Differences between time points per each antigen were assessed using Wilcoxon signed-rank test, *p < 0.05, and **p < 0.001

Long-term analysis of a sub-group of 24 *P. vivax* patients for which samples were available six months after enrolment showed a significant decrease of OD values only for MSP1 (OD average decrease of -43%, Day 0: 1.71 vs Day 180: 0.97, p < 0.05) and CSP (OD average decrease of -28%, Day 0: 0.83 vs Day 180: 0.60, p < 0.001). Nine PE antigens showed more stable IgG levels with an average decrease of between antigens of 6% (range: -2 to -21 and SD ± 6%) (Fig. 7). These subjects did not experience a new malaria episode during six months of follow-up by monthly microscopy confirmation, indicating that some PE antibodies are maintained for a significant period after initial infection in the absence of boosting.

### Human T cell responses

T cell responses were evaluated to eight antigens in a small set of individuals (n = 17) for which PMBCs were available. An ex vivo IFN-gamma response was detected in at least one subject for all the antigens tested. Positivity for IFN-gamma reactivity was the highest for the blood stage protein MSP1 and the pre-erythrocytic stage protein CSP, both at 35.3% (6/17 volunteers positive). From the remaining antigens tested, the highest percentage of positive responses was for ETRAMP at 33.3% (5/15), followed by Falstatin (25.0%, 4/16), CelTOS (23.5%, 4/17), the Hypothetical protein PVX_119755 (23.1%, 3/13) and HSP (18.2%, 2/11).

**Fig. 8:**
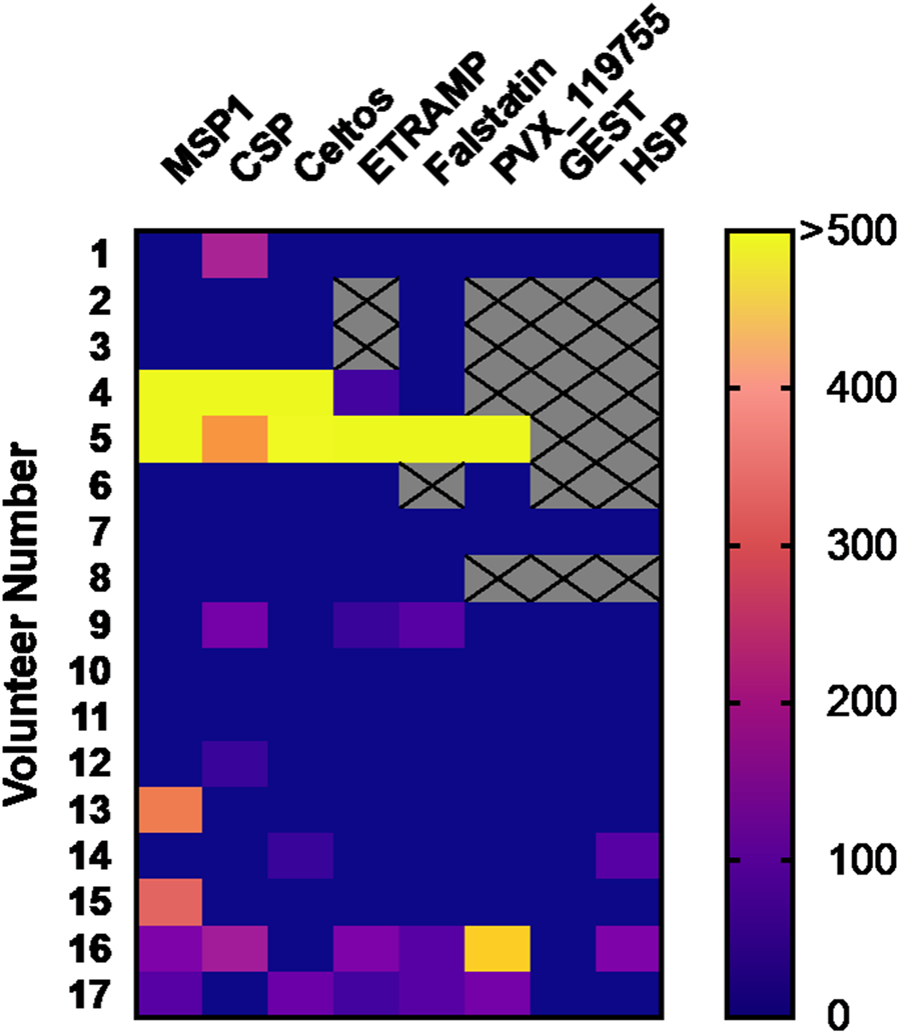
IFN-γ response to seven PE *P. vivax* antigens evaluated in 17 malaria patients from Iquitos. Shown as SFU/10^6^ PBMCs for samples which were > 20% above negative controls after subtracting background. Boxes with “X” indicate samples not measured due limited sample availability

Across individuals, broad and variable T cell responses to PE antigens was observed (Fig. 8) with two subjects positive to CSP only, and six subjects positive to 2–4 PE antigens (in addition to or besides CSP). Variability between subjects in the number of SFU (spot forming units) per million cells was also seen, with two subjects highly reactive to multiple antigens. Additionally, two subjects were positive to only MSP-1 and no T cell response was found for seven subjects (41%).

Antibody response was tested in seven of the subjects for which both plasma and PBMCs were available. In this limited set, there was no apparent correlation between positive IgG response and ex vivo IFN-gamma response (Table 4). Together, these data indicate that T cell responses to both blood stage and PE antigens are also present but variable during acute *P. vivax* infection.

**Table 4:** Comparative analysis of antibody and T cell responders against blood stage and PE *P. vivax* antigens

Protein ID	PVX Code	Parasite stage	ELISA +	ELISPOT +	IgG+ / IFN+	IgG+ / IFN-	IgG− / IFN+	IgG− / IFN-
MSP1	PVX_099980	Blood	100%	29%	2/7	5/7	0/7	0/7
CSP	PVX_119355	PE	86%	29%	2/7	4/7	0/7	1/7
CelTOS	PVX_123510	PE	57%	29%	2/7	2/7	0/7	3/7
ETRAMP	PVX_121950	PE	50%	33%	1/6	1/3	1/6	1/3
FALSTATIN	PVX_099035	PE	57%	14%	1/7	3/7	0/7	3/7
Hypothetical	PVX_119755	PE	80%	20%	1/5	3/5	0/5	1/5
HSP	PVX_089585	PE	100%	0%	0/1	1/1	0/1	0/1
GEST	PVX_121950	PE	100%	0%	0/1	1/1	0/1	0/1

Prevalence of ELISA and ELISPOT was measured by IgG and Interferon gamma positivity in *P. vivax* infected individuals, (n = 7)

## Discussion

Only a few *P. vivax* PE proteins such as CSP, TRAP and CelTOS have been thoroughly evaluated as vaccine candidates and still remain at the pre-clinical or early clinical stages [33]. More recently, have been seeing that natural acquired antibodies against *P. vivax* PE CelTOS, SPECT1, SSP3 and CSP proteins achieved significant inhibitory activity into formation of exoerythrocytic forms (EEFs) in in-vitro sporozoite-hepatocyte infection model, suggesting an important role of naturally acquired humoral immunity over the development of EEFs in *P. vivax* infected patients [34, 35].

New *P. vivax* PE candidates and, in particular, discovery of new antigens will be necessary for a better understanding of *P. vivax* PE immunity and to provide insight into their role in the prevention of both liver and blood stage infection. In the absence of well-established animal models of *P. vivax* infection, studying the acquisition of PE antibodies during natural infection is a logical starting point with the long-term goal of studying the role these PE antibodies play in functional immunity in order to justify integrating functional targets into novel vaccine candidates. In addition, as more candidates move into clinical trials in *P. vivax*-endemic areas, the level and impact of pre-existing immunity will be important to measure. However, to date, few studies of this kind been done for *P. vivax* PE antibodies.

A *P. vivax* cohort study (n = 31) in a low-transmission *P. vivax* area in western Thailand during 2013 showed prevalence of 76–86%, 18–21% and 0% for *Pv* CSP, CELTOS and TRAP antigens, respectively [36]. In addition, a cross-sectional study (n = 299) of naturally acquired immunity performed in Brazil during 2016 against whole protein and linear synthetic peptides of PE TRAP showed prevalence of 49% and 25–32%, respectively [37]. These results showed naturally acquired immunity against all 13 PE antigens tested in n = 76 *P. vivax* patients from a low endemic malaria region located in the Peruvian Amazon Basin was generally higher, with some individuals responding to all PE antigens (range of 58–99%). This variation could be related to the inherent characteristics of different circulating *P. vivax* parasites, differences in transmission dynamics or simply differences in protein production platforms as these previous studies used HEK-293 cell lines for production compared to wheat germ cell-free system.

Compared to the magnitude of *P. vivax* blood stage responses, the IgG response to PE antigens was lower. This is likely since boosting effect of anti-PE IgG with repeated infection was not observed. Rather, IgG responses to one antigen often correlated simply with a response to another antigen within individuals—leading to high and low antibody responders. Comparative analysis of high and low antibody responders against *P. vivax* PE and blood stage antigens vs. parasitaemia showed that high responders had lower parasitaemia levels. However, given the high correlation between antibody levels between both PE and blood stage antigens, correlations that may indicate if antibodies to any stage or target might be leading to protection against higher parasitaemia was not identified. These data suggest that high antibody levels may be a host-intrinsic factor, and this breadth of response—rather than a response to any single antigen—could be involved in the lower parasitaemia was observed. This aligns with published data demonstrating protection from *P. vivax* is associated with the acquisition of a multi-antigen antibody response at the blood stage [20]. However, low parasitaemia in high responders was seen irrespective of the number of previous *P. vivax* episodes, although these were not subsequent infections in the same individual. Thus, whether the lower parasitaemia is the result of a recently-acquired diverse antibody response better controlling subsequent infections or that the antibodies are a correlate of some other immune phenomenon that results in lower parasitaemia [38] will require investigation in well-designed longitudinal cohort studies with deeper immune phenotyping and active detection of new or relapse infections.

Previous studies have shown a correlation between antibody response and *P. vivax* parasitaemia levels in cross-sectional and cohort studies. This includes a large (n = 466) survey of the antibody response against blood stage protein vaccine target DBP-II which showed a negative correlation between *P. vivax* parasitaemia and antibodies in an area with hypo-endemic *P. vivax* transmission in Brazil [39]. As such, *P. vivax* DBP-II is the leading blood stage candidate for *P. vivax* blood stages. However, little is known about relationship between antibody response against PE antigens and parasitaemia in cross-sectional or cohort studies. Here, in a cross-sectional study in hypoendemic malaria area a modest negative correlation between parasitaemia and antibodies levels against 4 PE antigens was observed (Spearman RHO: -0.23 to -0.28, p < 0.05), suggesting these antigens should be included in further studies as functional antibody targets.

In addition, IgG antibodies at six months after *P. vivax* infection had variable stability, with a significant decrease in the most naturally immunogenic antibodies MSP-1(-43%) and CSP (-28%) antigens. However, nine other PE antigens tested showed relative stability (- 6% STD ± 6) as did the blood stage DBP antigen. This characteristic could be useful in defining serological markers of recent vs. historical exposure to use in malaria endemic areas near to effective malaria control or on way for malaria elimination process.

Even less well-studied than PE *P. vivax* antibodies are T cell responses to natural *P. vivax* infection. A few studies have assessed vaccine candidates eliciting CD8 + and/or CD4 + T cells that correlate with protection from liver-stage infection in mice [40, 41] and one clinical trial [42], but the mechanism that leads to T cell protection remains to be elucidated. Here, in a limited sample of volunteers, a positive T cell response in 35% vs 9–35% of tested individuals was observed against blood stage antigen MSP1 and PE antigens, respectively. From patients with a positive T cell response, 40% showed reactivity for both blood stage and PE antigens which did not appear to correlate with IgG responses. While the role of T cell-mediated protection against rodent malaria liver stages is robust [43, 44], the absence of appropriate animal models for *P. vivax* and, in particular, hypnozoites leaves this possibility unconfirmed. Interestingly, recent evidence demonstrated that CD8 + T cells can eliminate infected reticulocytes [45] opening the possibility of multi-stage CD8 + T cell vaccines to prevent or limit *P. vivax* infection. This limited data demonstrate that natural PE T cell immunity does exist and will need to be included in future studies with a larger population investigating the relationship between T cells and protection from infection at multiple stages.

## Conclusion

In conclusion, here is presented the most antigenically diverse survey of PE immunity acquired during natural infection using samples from people living in an endemic area of the Peruvian Amazon Basin. These results demonstrate broad but variable reactivity across *P. vivax* PE antigens and between individuals. Together, these data clearly demonstrate a previously underappreciated prevalence of natural immunity to PE antigens which warrants specifically designed longitudinal cohorts where better correlations to transmission and protection from infection can be assessed. Such studies can add to a growing understanding of immunity to *P. vivax* infection in terms of both antigens and immune effector mechanisms with the goal of designing vaccines capable of preventing or reducing infection in civilian and deployed military populations.

## Disclaimers

“The views expressed in this article reflect the results of research conducted by the author and do not necessarily reflect the official policy or position of the Department of the Navy, Department of Defense, nor the United States Government”

## Data Availability

All data generated or analyzed during this study are included in this published article.

## Abbreviations

PE: Pre-erythrocytic
ELISA: Enzyme-linked immunosorbent assay
MSP1: Merozoite surface protein-1
DBP: Duffy Binding Protein
CSP: Circumsporozoite protein
WHO: World Health Organization
TRAP: Thrombospondin-related adhesive protein
RH5: Reticulocyte-binding protein Homolog 5
RAS: Radiation attenuated sporozoites
GEST: Egress and sporozoite traversal
ETRAMP: Early transcribed membrane protein
STD: Standard deviation
PCR: Polymerase Chain Reaction
